# Bleaching Enamel

**Published:** 1903-08-15

**Authors:** Joseph Head


### BLEACHING ENAMEL.

BY JOSEPH HEAD, D.D.S.

Peroxide of hydrogen and peroxide of sodium when heated give off oxygen in great quantities. This nascent oxygen at the moment when it leaves the compound is most caustic, and, therefore, if we can liberate this gas directly on a tooth, we shall be able to remove any organic stain. Either pyrozone, which is twenty-five percent solution of peroxide hydrogen, or sodium dioxide can be used. These two materials seem equally powerful, but they are somewhat different in their actions, and, therefore, would better be described separately.

If the stained tooth is pulpless and the apical ends of the canals have been tightly sealed, the treatment should be as follows: Apply the rubber dam and tie one if not two ligatures around the neck, so that leakage is impossible. Let the rubber dam go slightly over the nostrils of the patient to prevent the fumes of the nascent oxygen from irritating the air passages. Oil should be rubbed on the hands of the operator and on the face of the patient. The tooth should be dried internally and externally. Cotton soaked in pyrozone should be packed in the canal, and a hot-ball burnisher, such as is used in plastic work, should be placed against the cotton, so that the steam of the nascent oxygen will be driven through the tooth substance. After the cotton becomes dry, it should be removed, and the tooth heated again internally with hot air, when the process described above can be repeated several times. Finally, cotton soaked in pyrozone should be sealed in the canal with hard gutta-percha, so that any gas that is given off may, by pressure, be driven throughout the dental tubules.

Then we are ready for the second stage of the process. The enamel should be thoroughly dried with hot-air blasts and heated instruments until the patient feels the heat of the tooth in the gum. This makes the oxygen within the tooth canal exert great pressure; then a piece of cotton soaked in pyrozone should be placed on the enamel, and a broad, hot instrument held against it, so that the steam shall be driven in from the outside. This should be continued until the cotton becomes dry, when the enamel should be ironed with a highly-heated ball burnisher. This drives out the pyrozone that the enamel has soaked up, and, in drying it out, liberates the nascent oxygen within the enamel substance. The effect of this last-mentioned ironing is most marked, and the stains can be seen perceptibly to whiten. This process can be repeated in its various stages as often as desirable, and when the patient leaves, fresh pyrozone should be sealed in the canal with gutta-percha, in order that the bleaching process may continue until the next visit. If, finally, there is a slight stain that the nascent oxygen will not remove, a strong solution of oxalic acid should be used in the same way. The oxalic acid is not only a powerful oxidizer of organic material, but will also change any iron stain to a colorless oxalate.

What has been said about the use of pyrozone, with a few cautions, applies equally well to peroxide of sodium, as both bleach primarily by setting free nascent oxygen. The peroxide of sodium is most valuable where oil in the tooth is to be saponified, and, therefore, it will sometimes succeed where pyrozone fails; however, we must remember that when peroxide of sodium touches the soft tissues, it makes a deep burn, which pyrozone would not, and, therefore, great care should always be used that the sodium dioxide does not escape from the protection of the rubber dam. When this compound is placed in hot water, the oxygen is given off so rapidly that a distinct puff is heard, but when it is melted on ice, a thick paste of the undischarged sodium dioxide can be obtained which when placed upon the dried enamel, and heated with a hot instrument will safely give off a tremendous quantity of nascent oxygen. The oxygen, as previously stated, will bleach the enamel of a tooth where the pulp is alive. When peroxide of sodium has been used it should be carefully washed off with water and neutralized with a weak acid. Before the rubber dam is removed, always be sure that none is left to burn the mouth. It is also, for the same reason, dangerous to seal it up in a tooth unless the utmost precautions are taken against its escape.—*International.*

[Caution is necessary in using this method, as both of these agents are explosive used in this way, and care should also be exercised to not overheat the tooth or the enamel will be chalked.—Ed.]

